# MD‐1 Deficiency Mitigates Atherosclerosis by Inhibiting NLRP3 Inflammasome Activation via cGAS‐STING Dependent Pathway in Macrophages

**DOI:** 10.1111/cpr.70288

**Published:** 2026-10-14

**Authors:** Fang Zhao, Zhili Jin, Zhiwei Cai, Quan Zhang, Tao He, Xiangjie Kong, Ming Chen, Zhibing Lu, Xiaorong Hu, Wen‐Lin Cheng

**Affiliations:** ^1^ Department of Cardiology, Hubei Provincial Clinical Research Center for Cardiovascular Interventional Diagnosis and Treatment Zhongnan Hospital, Wuhan University Wuhan China; ^2^ Institute of Myocardial Injury and Repair Wuhan University Wuhan China; ^3^ Department of Obstetrics and Gynecology Union Hospital, Tongji Medical College, Huazhong University of Science and Technology Wuhan China

**Keywords:** atherosclerosis, cGAS‐STING, macrophage polarisation, MD‐1, NLRP3 inflammasome

## Abstract

MD‐1 plays significant roles in various pathological processes closely associated with cardiovascular diseases through its functions in different cell types. However, the critical effects and molecular mechanisms mediated by MD‐1 in macrophages involved in the development of atherosclerosis remain elusive. In this study, we observed that the expression of MD‐1 was upregulated in atherosclerotic plaques derived from human carotid arteries, as analysed through the Gene Expression Omnibus database. Additionally, MD‐1 expression was elevated in the aortas of hyperlipidemic ApoE‐deficient mice and in oxidised low‐density lipoprotein‐stimulated bone marrow‐derived macrophages, as demonstrated by RT‐PCR and western blot analyses. Furthermore, MD‐1 expression exhibited strong co‐localisation with macrophages, as confirmed by double immunofluorescence staining. Notably, MD‐1^−/−^ApoE^−/−^ mice subjected to a high‐fat diet displayed a significant reduction in atherosclerotic plaques throughout the entire aorta, aortic root, and brachiocephalic arteries. Moreover, the plaques in MD‐1^−/−^ApoE^−/−^ mice were more stable, characterised by a decreased necrotic core size, reduced macrophage infiltration, and lower lipid deposition, alongside an increased accumulation of collagen and smooth muscle cells. Bone marrow transplantation from MD‐1^−/−^ApoE^−/−^ mice to ApoE^−/−^ mice markedly attenuated the development of atherosclerosis. This attenuation was associated with an enhanced anti‐inflammatory macrophage phenotype and a reduction in pro‐inflammatory macrophage polarisation. Similar effects of MD‐1 ablation on macrophage behaviour were also observed in vitro. Mechanistically, MD‐1 deficiency inhibited the cyclic GMP‐AMP synthase‐stimulator of interferon genes‐TANK‐binding kinase 1‐IRF3 pathway, thereby attenuating NLRP3 inflammasome activation in macrophages. However, the use of the STING agonist DMXAA counteracted the effects of MD‐1 deficiency on macrophage polarisation. Our findings reveal MD‐1 promotes pro‐inflammatory (M1‐like) macrophage polarisation in atherosclerosis by inhibiting NLRP3 inflammasome activation via a cGAS‐STING dependent pathway. These results suggest that MD‐1 neutralisation could be a potential therapeutic strategy for atherosclerosis management.

## Graphical abstract

MD‐1 is induced and activated in macrophages. The activated MD‐1 subsequently promotes the cGAS‐STING‐TBK1‐IRF3 signalling pathway to activate the NLRP3 inflammasome, and subsequently increases pro‐inflammatory macrophage polarisation and decreases plaque stability to accelerate the development of atherosclerosis.

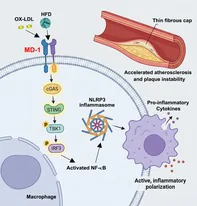

## 1 Introduction

Atherosclerosis is widely recognised as a chronic inflammatory disease [1] and is identified as the predominant pathological process underlying various cardiovascular diseases, including coronary heart disease, myocardial infarction, ischemic cardiomyopathy‐induced heart failure, ischemic stroke, peripheral vascular disease, and sudden cardiac death [2]. Collectively, these conditions represent the leading causes of morbidity and mortality worldwide [3]. During the progression of atherogenesis, circulating monocytes migrate and roll into the arterial sub‐endothelium, a process triggered by endothelial dysfunction, where they differentiate into macrophages. These macrophages play a crucial role in regulating the inflammatory response and engulf oxidised low‐density lipoprotein (OX‐LDL), transforming into lipid‐rich foam cells [4, 5]. Concurrently, defective phagocytic clearance of apoptotic macrophages leads to the formation of a necrotic core, which promotes the development of vulnerable plaques that can eventually disrupt and cause advanced plaque rupture [6]. Notably, accumulating evidence suggests that the switching of macrophage polarisation‐mediated inflammation is essential for the progression of atherosclerotic plaques. Classically activated pro‐inflammatory M1 macrophages initiate and sustain inflammation, while alternatively activated anti‐inflammatory M2 macrophages serve to inhibit it [7, 8]. Therefore, it is imperative to investigate the potential regulators and underlying mechanisms that modulate macrophage polarisation in the development of atherosclerosis, as well as to propose promising therapeutic strategies for its prevention.

Myeloid differentiation protein 1 (MD‐1, also termed Ly86) is a secreted glycoprotein that binds the TLR family member RP105, which is predominantly expressed in macrophages, B cells, dendritic cells, and other immune cells [9, 10]. Accumulating evidence has demonstrated that MD‐1 plays vital roles in multiple biological processes, including cell apoptosis, humoral immune regulation, obesity development, insulin resistance, and transmembrane signal transduction [11, 12]. Given its expression in cardiac tissues and upregulated levels in ligated and atherosclerotic carotid arteries, MD‐1 has recently been identified as a critical regulator of various cardiovascular diseases [13, 14]. Functionally, MD‐1 is well characterised to participate in inflammatory responses, immune activation, and multiple cardiovascular pathological processes. Specifically, MD‐1 deficiency facilitates left ventricular structural and electrical remodelling via the inactivation of the mitogen‐activated protein kinase (MAPK) and nuclear factor kappa B (NF‐κB) signalling pathways [15], whereas MD‐1 overexpression attenuates pathological cardiac remodelling by inhibiting the TLR4/STAT3 pathway to suppress myocardial oxidative stress and inflammation [16]. Loss of MD‐1 exacerbates myocardial ischemia/reperfusion (I/R) injury and increases ventricular arrhythmia susceptibility through TLR4/NF‐κB signalling activation [17]. Furthermore, MD‐1 knockdown diminishes intracellular lipid accumulation and endoplasmic reticulum oxidative stress in macrophages by modulating the SREBP2/HMGCR signalling pathway [18]. Nevertheless, the specific role of MD‐1 in modulating macrophage polarisation, as well as its direct contribution to the progression and stability of atherosclerotic plaques, remains largely unclarified.

In the current study, we observed a significant increase in MD‐1 expression within atherosclerotic tissues, predominantly in macrophages. Through the application of global gene knockout and bone marrow transplantation strategies, we elucidated the critical role of macrophage‐derived MD‐1 in macrophage polarisation, inflammation, and the progression of atherosclerosis. Mechanistically, we hypothesise that MD‐1 deficiency mitigated pro‐inflammatory macrophage polarisation by inactivating the NLRP3 inflammasome, which was dependent on the downregulation of the cGAS‐STING‐TBK1‐IRF3 signalling pathway. Furthermore, treatment with the STING agonist DMXAA effectively neutralised the anti‐inflammatory macrophage phenotype induced by MD‐1 deficiency. Our study offers novel insights into the role of macrophage‐derived MD‐1 in the context of atherosclerosis.

## 2 Materials and Methods

### 2.1 Animals and Treatment

Animal care and experimental procedures received approval from the Animal Care and Use Committee of Zhongnan Hospital and the Animal Experiment Center of Wuhan University, and all animal operations complied with the ARRIVE guidelines. Male and female MD‐1 heterozygous mice (8–10 weeks old) on a C57BL/6 background were procured from RIKEN (RBRC02386) and subsequently backcrossed to obtain stable MD‐1 homozygous knockout (MD‐1^−/−^) mice. Subsequently, MD‐1 mice were crossbred with apolipoprotein E‐deficient (ApoE^−/−^) mice to generate double‐knockout MD‐1 ApoE mice and littermate control ApoE mice. Both male MD‐1^−/−^ApoE^−/−^ and ApoE^−/−^ mice were maintained under a standard 12 h light/12 h dark cycle and were fed either a high‐fat diet (HFD) or normal chow (NC) for a duration of up to 28 weeks to facilitate the induction of atherosclerosis.

### 2.2 Human Samples

Atherosclerotic coronary artery plaque specimens were collected from patients with confirmed coronary heart disease who underwent heart transplantation. Written informed consent was obtained from the respective families. All procedures involving human samples adhered to the principles outlined in the Declaration of Helsinki and received approval from the Institutional Review Boards of Renmin Hospital of Wuhan University and Zhongnan Hospital of Wuhan University (approval no. 2025058K) (Wuhan, China).

### 2.3 Bone Marrow Transplantationt

Eight to 10‐week‐old male MD‐1^−/−^ApoE^−/−^ and ApoE^−/−^ mice were utilised as donors, while age‐matched ApoE^−/−^ mice served as recipients. On the day of transplantation, recipient mice received lethal whole‐body irradiation with two doses of 550 rads (total 11 Gy), with a 4‐h interval between the two irradiations. Following irradiation, the mice were anaesthetised intraperitoneally with pentobarbital sodium at a dosage of 50 mg/kg. Bone marrow nucleated cells were isolated from the femurs and tibiae of donor mice, and approximately ~5 × 10^7^ viable nucleated bone marrow cells were injected into recipient ApoE mice via the tail vein to construct two chimeric mouse models: MD‐1^−/−^ApoE^−/−^ → ApoE^−/−^ and ApoE^−/−^ → ApoE^−/−^ mice. After 4 weeks on a normal chow diet post‐transplantation, the chimeric mice were subsequently fed a high‐fat diet (HFD) for an additional 16 weeks to induce atherosclerosis.

### 2.4 Atherosclerotic Plaque Analysis

After 28 weeks of dietary intervention, mice were euthanised by intraperitoneal injection of excess pentobarbital sodium. The thoracic and abdominal cavities were quickly opened, and mice were perfused transcardially with phosphate‐buffered saline (PBS) to flush out intravascular blood, followed by 4% paraformaldehyde for tissue fixation. The entire aorta (from the aortic root to the iliac artery bifurcation) was carefully and completely dissected under a stereomicroscope, with surrounding adipose tissue and connective tissue thoroughly removed to avoid interference. Dissected aortas were longitudinally cut open along the vascular wall, flattened and fixed on filter paper, then rinsed with 60% isopropanol for 2 min. Subsequently, aortas were stained with Oil Red O working solution at room temperature for 1 h in the dark. After staining, excess dye was washed away with 60% ethanol until the background was clear, and the aortas were rinsed with distilled water and flattened for imaging. High‐resolution macroscopic images were captured using a stereo fluorescence microscope (OLYMPUS SZX16). Plaque area quantification was performed using Image Pro Plus 6.0 software. The positive Oil Red O‐stained area (red lipid plaque area) and the total aortic surface area of each sample were measured. Hearts containing complete aortic root tissues were isolated, partially embedded in paraffin and partially embedded in optimal cutting temperature (OCT) compound. Continuous 5 μm‐thick frozen sections were prepared at the atrioventricular valve level. Haematoxylin and Eosin (H&E) staining and Oil Red O staining were performed to observe plaque morphological structure and lipid deposition. Plaque area, necrotic core area and lipid deposition area were quantitatively analysed by Image Pro Plus 6.0 software with consistent field‐of‐view and threshold standards.

### 2.5 Immunofluorescence Staining

Aortic root sections (5 μm thick) from experimental mice underwent a 5‐min high‐pressure antigen retrieval process. The sections were subsequently blocked in PBS containing 10% goat serum for 1 h at room temperature, followed by overnight incubation with primary antibodies at 4°C. After three washes with PBS, the sections were incubated with appropriate secondary antibodies at 37°C for 1 h. Nuclei were counterstained with 4′,6‐diamidino‐2‐phenylindole (DAPI). Images were captured using a fluorescence microscope (OLYMPUS DX51) and analysed with DP2‐BSW software.

### 2.6 Quantitative Real‐Time PCR and Western Blotting

Total RNA was isolated from mouse arterial tissues or cells using TRIzol reagent (ELK Biotechnology, cat# EP013) and subsequently purified with DNase. Complementary DNA (cDNA) was synthesised from the isolated RNA using a Transcriptor First Strand cDNA Synthesis Kit (ELK Biotechnology, cat# EQUATION 003). The expression levels of target genes were quantified via real‐time PCR employing the LightCycler 480 SYBR Green 1 Master Mix on a LightCycler 480 QPCR System (Roche Diagnostics, Indianapolis, IN). The mRNA levels were normalised against reference housekeeping genes to ensure accuracy. Tissue and cellular total protein were extracted using RIPA lysis buffer, and their concentrations were determined using a BCA (ASPEN, cat# AS1086) assay. Following centrifugation of the lysates, supernatants were subjected to fractionation via 10% SDS‐PAGE and subsequently transferred to PVDF membranes (ASPEN, cat# AS1012). The membranes were blocked in Tris‐buffered saline (TBS) containing 5% nonfat milk for 1 h at room temperature, followed by overnight incubation with primary antibodies at 4°C: MD‐1 (Invitrogen, cat# PA5‐20261), iNOs (Wuhan Sanying, cat# 18985‐1‐AP), IL‐6 (abclonal, cat# A0286), IL‐10 (abclonal, cat# A2171), PPARγ (Wuhan Sanying, cat# 16643‐1‐AP), GAPDH (abcam, ab181602), cGAS (Wuhan Sanying, cat# 84045‐1‐RR), p‐STING (CST, cat# 72971), STING (Wuhan Sanying, cat# 19851‐1‐AP), p‐TBK1 (CST, cat# 5483), TBK1 (CST, cat# 3504), p‐IRF3 (CST, cat# 29047), IRF3 (CST, cat# 4302), p‐NF‐κB p65 (CST, cat# 303), NF‐κB p65 (Wuhan Sanying, 10745‐1‐AP), pro IL‐1β (CST, cat# 12242), IL‐1β (santa, cat# sc‐12742), Caspase1 p10 (affbiotech, cat# AF4022). Afterward, the membranes were incubated with secondary antibodies, treated with enhanced chemiluminescence reagents, and visualised using a FluorChem E Imager (ProteinSimple, San Jose, CA).

### 2.7 BMDM Isolation and Treatment

The bilateral femurs and tibiae were quickly isolated from MD‐1^−/−^ApoE^−/−^ and ApoE^−/−^ mice under sterile conditions. The muscle and connective tissues attached to the bone surface were completely removed, and both ends of the bones were cut off to expose the bone marrow cavity. Pre‐cooled sterile RPMI 1640 complete medium was used to flush the bone marrow cavity repeatedly until the bone cavity turned white. The flushed bone marrow cell suspension was collected and filtered through a 70 μm sterile cell strainer to remove tissue debris. The filtered cell suspension was centrifuged at 1000 rpm for 5 min, the supernatant was discarded, and the cell pellet was resuspended in RPMI 1640 medium (Gibco, cat# 11875093) supplemented with 10% foetal bovine serum and 50 ng/mL macrophage colony‐stimulating factor (M‐CSF, PeproTech, cat# 315‐02). Cells were seeded in cell culture plates at a density of 7 × 10^5^ cells per plate and cultured in a 37°C, 5% CO_2_ incubator. The culture medium was refreshed every 3 days, and mature BMDMs were obtained after 7 days of continuous induction and differentiation. To establish an in vitro atherosclerosis model, mature BMDMs were treated with 50 μg/mL OX‐LDL (Yeasen, cat# 20605ES05) for 24 h to induce foam cell formation and inflammatory response. For further investigation of the regulatory role of the cGAS‐STING pathway in MD‐1‐mediated macrophage polarisation, BMDMs were treated with 150 μg/mL DMXAA (a canonical STING agonist; Sigma, cat# D5817) for 24 h for functional verification.

### 2.8 Foam Cell Formation

BMDMs were cultured on chamber slides for overnight incubation. Following a 24‐h serum starvation, the cells were treated with OX‐LDL at a concentration of 50 μg/mL for an additional 24 h. After fixation with 4% paraformaldehyde, the cells were stained with Oil Red O and assessed using microscopy. For quantification purposes, the cells were subsequently incubated with isopropanol to elute the Oil Red O dye from the cells.

### 2.9 Measurement of Inflammatory Cytokines

Mouse serum levels of Tumour Necrosis Factor‐alpha (TNF‐α), Interleukin‐6 (IL‐6), Mannose Receptor C‐Type 1 (Mrc1), and Interleukin‐10 (IL‐10) were measured by ELISA kits according to their manufacturer's instructions. The kits used were: mouse TNF‐α ELISA Kit (Invitrogen, cat# BMS607‐3TEN), mouse IL‐6 ELISA Kit (Invitrogen, cat# BMS213‐2TEN), mouse Mrc1 ELISA Kit (ELK Biotechnology, cat# ELK3454), mouse IL‐10 ELISA Kit (Invitrogen, cat# BMS614TEN). Absorbance at 450 nm was measured in each well by visualisation of colour intensity development.

### 2.10 Flow Cytometry

Peripheral blood serum samples from MD‐1^−/−^ApoE^−/−^ → ApoE^−/−^ mice and ApoE^−/−^ → ApoE^−/−^ mice were incubated at 4°C for 30 min with the following labelled antibodies: FITC anti‐mouse CD11b antibody (Invitrogen, 11–0112‐81), APC anti‐mouse CD115 antibody (Invitrogen, 17–1152‐82), and PE anti‐mouse Ly‐6C antibody (Invitrogen, 12–5931‐82) in accordance with the manufacturer's instructions. Subsequently, the cells were washed three times with PBS and analysed using a Beckman CytoFLEX cytometer.

### 2.11 RNA Sequencing

BMDMs were isolated from the femurs and tibias of MD‐1^−/−^ApoE^−/−^ and ApoE^−/−^ mice, and subsequently stimulated with OX‐LDL at a concentration of 50 μg/mL for 24 h. Following stimulation, transcriptome sequencing was performed. Total RNA was extracted using the RNA‐easy Isolation Reagent (Vazyme, China), and the quality and quantity of RNA were assessed using a NanoDrop 2000 spectrophotometer. Differential expression analysis was performed using the DESeq2 package in R, considering genes showing a false discovery rate (FDR) < 0.05 and a fold change (FC) ≥ 1 as significantly differentially expressed. Functional enrichment analysis was performed through Gene Ontology (GO) and KEGG pathway analyses to elucidate the biological implications of the findings.

### 2.12 Co‐Immunoprecipitation

For immunoprecipitation, cells were washed with cold PBS and lysed with lysis buffer containing Protease Inhibitor Cocktail Tablets (ASPEN, cat# AS1005C) and centrifuged at 13,000 × *g* for 15 min, and the supernatant was collected as total protein lysate. After being pre‐cleared with homologous normal immunoglobulin G and protein A/G‐agarose beads (BIO‐RAD, cat# 1614813), lysates were incubated with the indicated primary antibodies and Protein G‐agarose at 4°C overnight with gentle shaking. The immunoprecipitated proteins were further washed five times with lysis buffer, boiled with 2× SDS loading buffer, separated with SDS‐PAGE and electrophoretically transferred to polyvinylidene difluoride membranes (Millipore). The membranes were blocked with 5% BSA in Tris‐buffered saline containing 0.1% Tween‐20 and were immunoreacted with the indicated primary antibodies and secondary antibodies conjugated to HRP.

### 2.13 Statistical Analysis

Data were analysed using SPSS software (version 19.0) and are presented as mean ± standard deviation (SD). Comparisons between two groups were conducted using two‐tailed Student's *t*‐tests or Wilcoxon rank‐sum tests, depending on the normality of the data distribution. For comparisons among more than two groups, one‐way ANOVA was employed. A *p* value of less than 0.05 was deemed statistically significant.

## 3 Results

### 3.1 MD‐1 Is Increased in Atherosclerotic Tissues and Is Primarily Localised in Macrophages

To investigate the association between MD‐1 and the development of atherosclerosis, we first analysed its expression in human samples. We utilised publicly available datasets from the Gene Expression Omnibus (GEO) database (accessions GSE28829 and GSE43292), which include patients with macroscopically visible or atheroma plaques, as well as early and advanced atherosclerotic plaques (Figure 1A). We particularly focused on candidates that exhibited consistent trends across these datasets (Figure 1B). The results revealed that MD‐1 expression was significantly upregulated in advanced atherosclerotic plaques compared to early‐stage plaques (Figure 1C). A similar trend was noted when comparing atheromatous plaques to macroscopically normal tissue (Figure 1D). In mouse and cellular models, MD‐1 mRNA and protein levels were elevated in the aortas of hyperlipidemic ApoE^−/−^ mice fed a HFD compared to those on NC for 28 weeks (Figure 1E). Furthermore, MD‐1 exhibited time‐dependent increases in bone marrow‐derived macrophages (BMDMs) following OX‐LDL stimulation (Figure 1F). Immunofluorescence staining confirmed that MD‐1 colocalises primarily with macrophages in both OX‐LDL‐stimulated BMDMs and mouse atherosclerotic plaques (Figure 1G,H). Collectively, these findings suggest that elevated MD‐1 in macrophages may play a significant role in the development of atherosclerosis.

**FIGURE 1:**
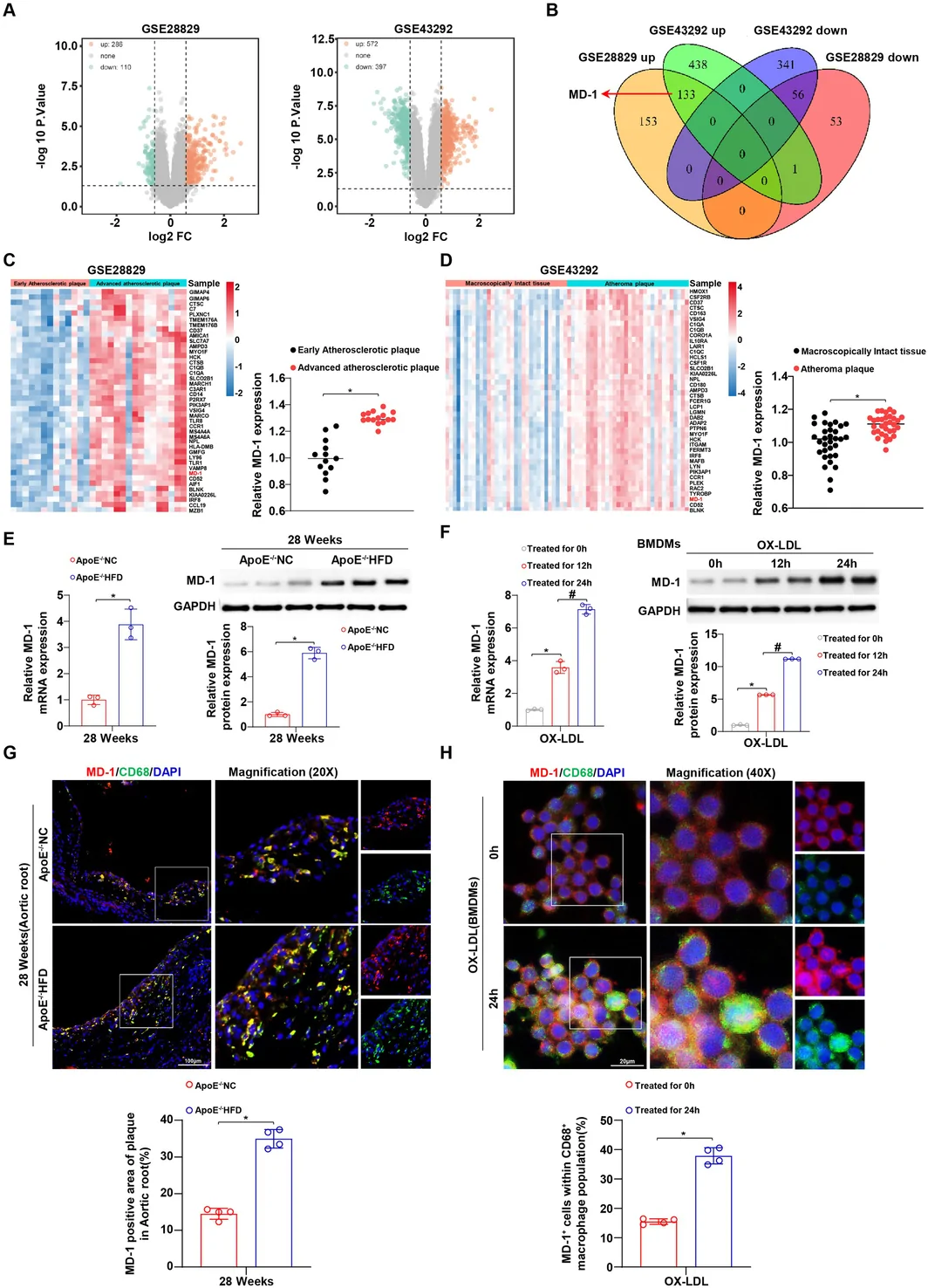
Elevated MD‐1 expression in patients and mice with atherosclerotic plaque. (A) Volcano plot of DEGs from GSE28829 and GSE43292 datasets. (B) Venn diagram of overlapping DEGs between GSE28829 and GSE43292. (C) Heatmap of gene expression in early versus advanced atherosclerotic plaques (GSE28829). *p < 0.05: Advanced versus early plaque. (D) Heatmap of gene expression in macroscopically normal versus atheromatous plaques (GSE43292). *p < 0.05: Atheroma plaque versus macroscopically normal tissue. (E) MD‐1 mRNA and protein levels in aortas of ApoE^−/−^ mice fed normal chow (NC) or high‐fat diet (HFD) for 28 weeks. *n* = 3. *p < 0.05: HFD versus NC group. (F) MD‐1 mRNA and protein levels in BMDMs treated with 50 μg/mL OX‐LDL at indicated times. *n* = 3. *p < 0.05: OX‐LDL stimulated 12 h versus unstimulated group; ^#^ *p* < 0.05: OX‐LDL stimulated 24 h versus 12 h group. (G) Representative images of MD‐1 (red) colocalisation with CD68+ macrophages (green) in plaques of aortic root from ApoE^−/−^ mice fed NC or HFD for 28 weeks. *n* = 4. Scale bar: 100 μm. (H) Representative immunofluorescence images showing MD‐1 (red) colocalisation with CD68+ macrophages (green) in OX‐LDL‐treated BMDMs. Nuclei counterstained with DAPI (blue). *n* = 4. Scale bar: 20 μm.

### 3.2 MD‐1 Knockout Inhibits Atherogenesis In Vivo

To determine whether the significant alteration of MD‐1 in atherosclerotic tissues from humans and mice could influence the development of atherosclerosis, we employed a loss‐of‐function strategy by crossing MD‐1 deficient mice with ApoE^−/−^ mice (MD‐1^−/−^ApoE^−/−^) and their ApoE^−/−^ littermates in vivo. As demonstrated in Figure 2A,B, both groups fed with HFD for 28 weeks exhibited a significant increase in plaque formation across the entire aorta, as assessed by en face analysis stained with Oil Red O. Notably, the MD‐1^−/−^ApoE^−/−^ mice displayed a more pronounced reduction in plaque formation (16.35%) compared to the ApoE^−/−^ group (41.79%), although no differences were observed between the two groups under normal chow (NC) treatment (Figure 2A,B). Furthermore, plaque formation in the brachiocephalic arteries and aortic root sections was evaluated through HE staining to further elucidate the role of MD‐1 in atherogenesis in mice. The results indicated that MD‐1^−/−^ApoE^−/−^ mice exhibited a significant reduction in plaque area in both the brachiocephalic arteries (Figure 2C,D) and the aortic root (Figure 2E,F) when stimulated by HFD, with only slight changes observed in the NC‐treated groups. Additionally, there were no significant differences between the MD‐1^−/−^ApoE^−/−^ and ApoE^−/−^ groups under HFD treatment regarding serum parameters, including triglycerides, plasma cholesterol levels, or lipoprotein profiles (very low‐density lipoprotein, intermediate‐density lipoprotein, LDL, and high‐density lipoprotein) (Figure 2G).

**FIGURE 2:**
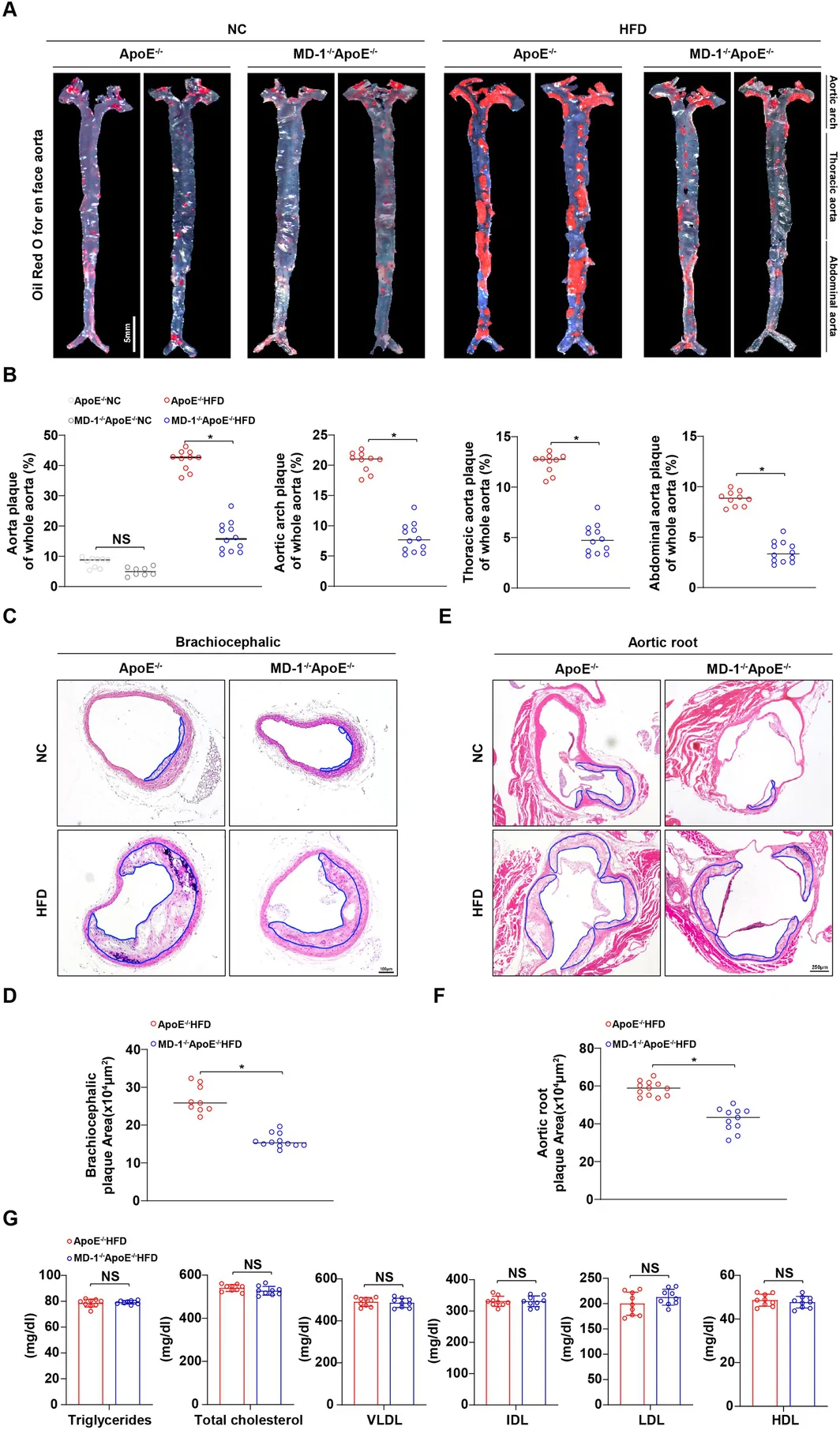
Attenuated atherosclerotic plaque area in HFD‐fed MD‐1^−/−^ApoE^−/−^ mice. (A, B) En face Oil Red O staining of aortas from MD‐1^−/−^ApoE^−/−^ and ApoE^−/−^ mice fed with NC or HFD for 28 weeks. The bottom panel showed the quantification of the oil red O‐positive area in the en face aorta. *n* = 8–12. Scale bar: 5 mm. (C, D) Brachiocephalic artery sections from MD‐1^−/−^ApoE^−/−^ and ApoE^−/−^ mice fed with NC or HFD were stained with haematoxylin and eosin. *n* = 9–12. Scale bar: 100 μm. (E, F) Aortic root sections from MD‐1^−/−^ApoE^−/−^ and ApoE^−/−^ mice fed with NC or HFD were haematoxylin and eosin stained. *n* = 11–12. Scale bar: 250 μm. (G) Elisa analysis of triglycerides, total cholesterol, and lipoprotein profiles of serum from MD‐1^−/−^ApoE^−/−^ and ApoE^−/−^ fed with HFD. *n* = 9. *p < 0.05 versus ApoE^−/−^ mice treated with HFD group.

### 3.3 MD‐1 Ablation Promotes Plaque Stability

The features of vulnerable plaques, resulting from specific changes in cellular composition, are more critical for the occurrence of severe clinical cardiovascular outcomes than plaque volume. This includes factors such as the size of the necrotic core, deposition of collagen, smooth muscle cells, macrophages, and lipids. As expected, the plaque composition in MD‐1^−/−^ApoE^−/−^ mice exhibited an anti‐vulnerable phenotype, characterised by a reduced size of necrotic cores and an increased fibrous cap cover in the brachiocephalic arteries and aortic root when compared to ApoE^−/−^ mice (Figure 3A,B). MD‐1^−/−^ApoE^−/−^ mice demonstrated a significant decrease in subintimal lipid content, with a robust 46.3% reduction in lipid deposits in plaques stained with Oil Red O compared to controls (Figure 3C). Moreover, the infiltration of macrophages, identified by CD68 positive regions, was reduced by 43.2% in the plaques of MD‐1^−/−^ApoE^−/−^ mice compared to ApoE^−/−^ mice (Figure 3D). In contrast, the collagen‐positive area, as indicated by Picrosirius Red (PSR) staining, and smooth muscle cell (SMC) content, as shown by smooth muscle actin (α‐SMA) staining, in atherosclerotic plaques were significantly increased in MD‐1^−/−^ApoE^−/−^ mice relative to controls, with a 33% increase in PSR (Figure 3E) and a 47.1% increase in α‐SMA positive area (Figure 3F) within the plaques, respectively. Collectively, MD‐1 deficiency remarkably enhances plaque stability.

**FIGURE 3:**
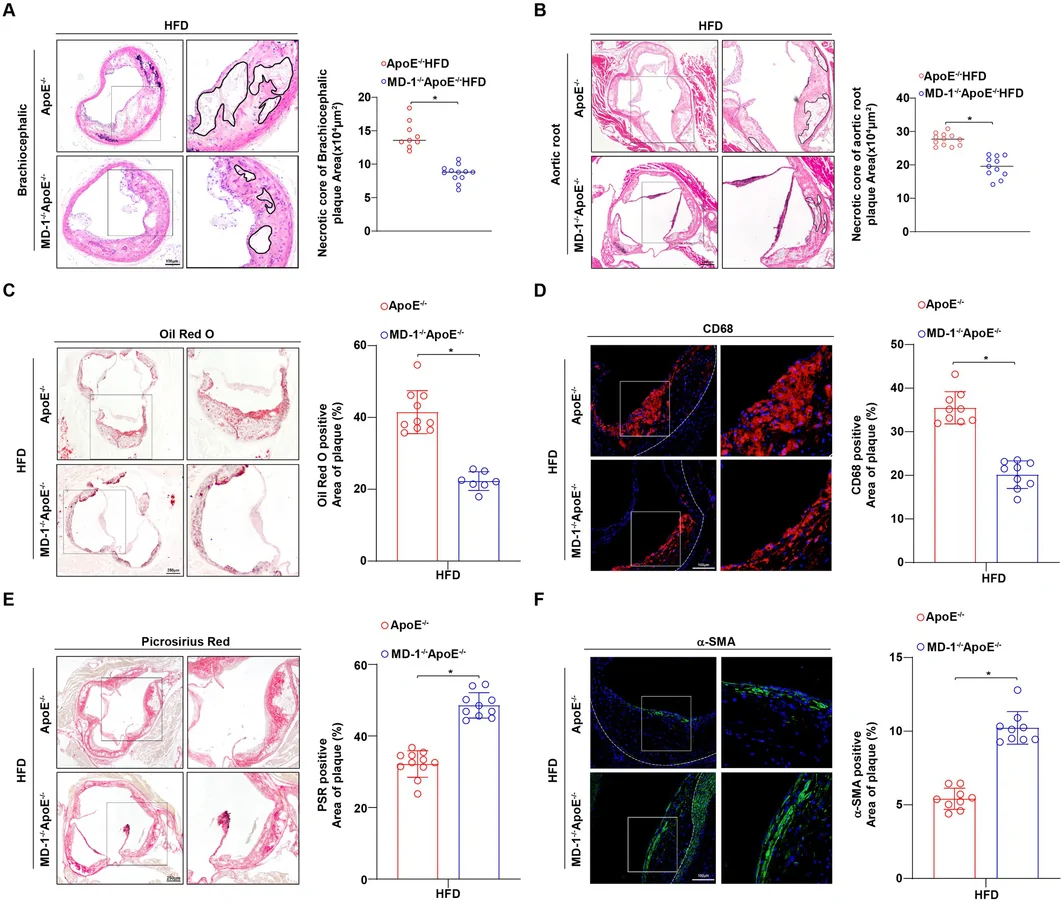
The necrotic area and plaque stability characteristics in MD‐1‐deficient mice. (A, B) Representative brachiocephalic artery sections (A) and aortic root sections (B) from MD‐1^−/−^ApoE^−/−^ and ApoE^−/−^ mice were stained with haematoxylin and eosin. The circular region indicates necrotic areas. *n* = 8–12. Scale bar: 100 μm (A). Scale bar: 250 μm (B). (C, D) Cross sections of the aortic root were stained with Oil Red O for lipids (*n* = 7–10), CD68 for macrophages (*n* = 9). Scale bar: 250 μm (C). Scale bar: 100 μm (D). (E, F) Cross sections of the aortic root were stained with picrosirius red for collagen (*n* = 10–11), α‐smooth muscle actin for smooth muscle cells (*n* = 9). *p < 0.05 versus ApoE^−/−^ mice treated with HFD group. Scale bar: 250 μm (E). Scale bar: 100 μm (F).

### 3.4 Macrophage‐Derived MD‐1 Deficiency Inhibits Atherosclerotic Plaque Formation

Given the elevated expression of MD‐1 during atherogenesis, particularly its primary localisation within macrophages, we reconstituted ApoE^−/−^ mice (recipient mice) with either MD‐1^−/−^ApoE^−/−^ or ApoE^−/−^ BMDMs via bone marrow transplantation to investigate whether the ablation of MD‐1 in haematopoietic cells predominantly contributes to the athero‐protective effect. Lethally irradiated recipients were subsequently maintained on a high‐fat diet (HFD) for 16 weeks to induce atherosclerosis (Figure 4A). Successful engraftment was confirmed by genomic PCR of circulating leukocytes (Figure 4B). En face analysis of Oil Red O‐stained aortas revealed significantly reduced atherosclerotic plaque formation in ApoE^−/−^ recipients transplanted with MD‐1^−/−^ApoE^−/−^ bone marrow (MD‐1^−/−^ApoE^−/−^ → ApoE^−/−^) compared to ApoE^−/−^ → ApoE^−/−^ controls (Figure 4C,D). Similarly, a marked reduction in aortic root plaque area was observed in H&E‐stained sections from MD‐1^−/−^ApoE^−/−^ → ApoE^−/−^ mice (Figure 4E,F). Consistently, serum levels of pro‐inflammatory markers (TNF‐α and IL‐6) were downregulated, while anti‐inflammatory cytokines (Mrc1 and IL‐10) were upregulated in MD‐1^−/−^ApoE^−/−^ → ApoE^−/−^ chimeras relative to controls (Figure 4G). Collectively, these findings, combined with results from global knockout and chimera models, demonstrate that macrophage‐specific loss‐of‐function of MD‐1 significantly attenuates atherogenesis.

**FIGURE 4:**
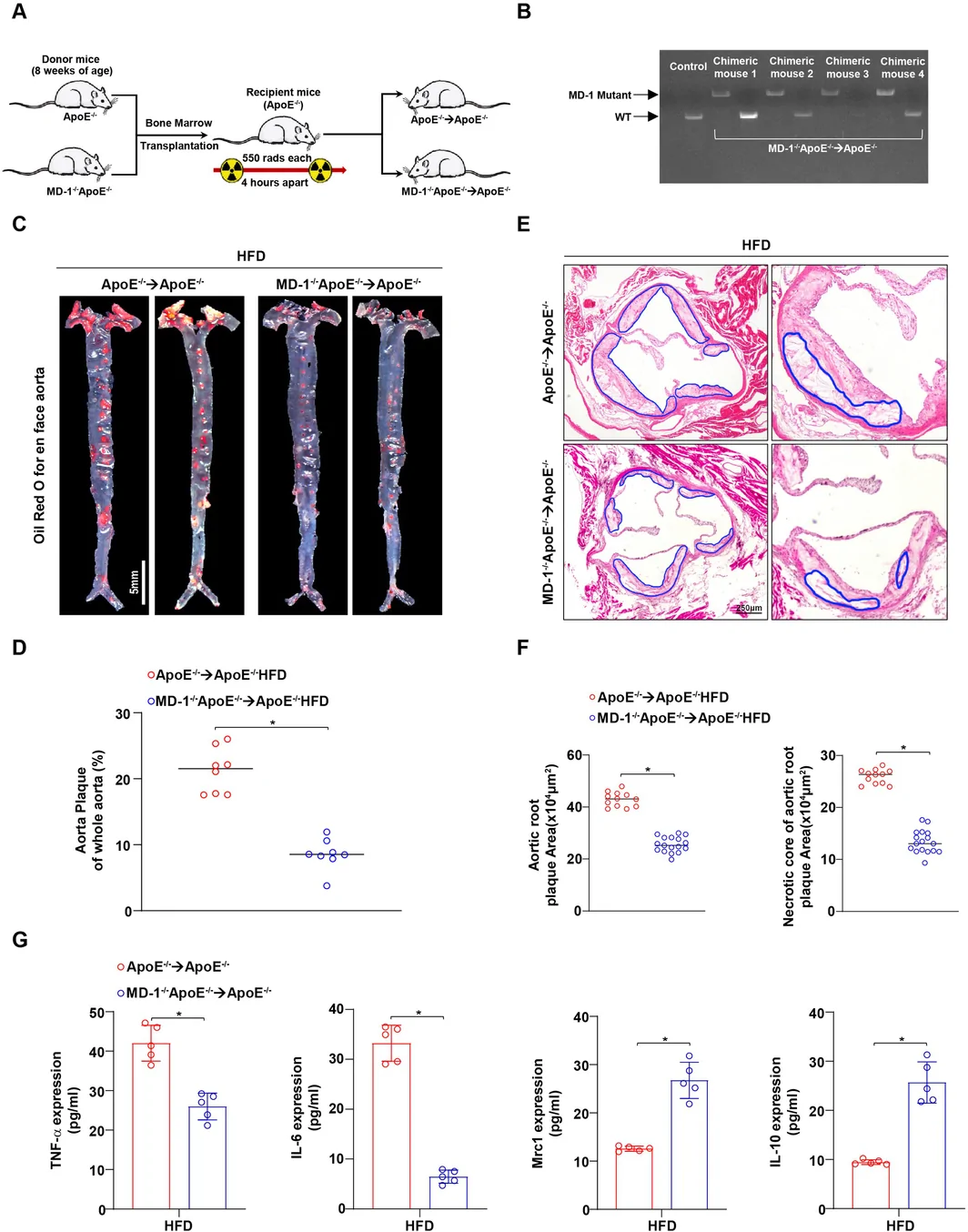
Macrophage‐derived MD‐1 contributes to atherosclerotic plaque development. (A) Schematic diagram showing the strategy of the bone marrow transplantation (BMT) experiment. ApoE^−/−^ mice were exposed to irradiation and then received bone marrow cells from male MD‐1^−/−^ApoE^−/−^ or ApoE^−/−^ mice via tail vein injection. (B) The genotype of WT and MD‐1 deletion in genomic DNA isolated from circulating white blood cells of recipient animals transplanted with MD‐1^−/−^ApoE^−/−^ bone marrow. (C, D) En face oil red O staining of aortas from MD‐1^−/−^ApoE^−/−^ → ApoE^−/−^ or ApoE^−/−^ → ApoE^−/−^ mice fed with a HFD for 16 weeks. The bottom panel showed the quantification of the oil red O‐positive area in the en face aorta. *n* = 8. Scale bar: 5 mm. (E, F) HE staining of aortic roots from MD‐1^−/−^ApoE^−/−^ → ApoE^−/−^ and ApoE^−/−^ → ApoE^−/−^ mice fed with a HFD for 16 weeks. The bottom panel showed quantification of plaque occupation or necrotic areas in mice that received bone marrow cells from MD‐1^−/−^ApoE^−/−^ or ApoE^−/−^mice. *n* = 12–17. Scale bar: 250 μm. (G) The TNF‐α, IL‐6, Mrc1 and IL‐10 levels in serum from MD‐1^−/−^ApoE^−/−^ → ApoE^−/−^ and ApoE^−/−^ → ApoE^−/−^ mice were measured by ELISA. *n* = 5. *p < 0.05 versus ApoE^−/−^ → ApoE^−/−^ mice treated with HFD group.

### 3.5 Macrophage‐Derived MD‐1 Attenuates Pro‐Inflammatory Polarisation in Atherosclerotic Plaques

Chronic inflammation is well known as a prominent feature of atherosclerosis; we next investigated whether this important pathological process was responsible for the effects of MD‐1 deficiency in macrophages on atherogenesis. The effect of MD‐1 on the number of subsets of circulating classical monocytes that differentiated into pro‐ or anti‐inflammatory macrophage phenotypes was firstly examined [19]. Total monocytes were first gated from live cells using the CD11b + CD115+ parent population. Subsequently, the CD11b + monocytes were further subdivided into Ly6C‐high and Ly6C‐low monocyte subsets based on Ly6C expression. Consistent with this gating hierarchy, we observed a decreased proportion of CD115 + Ly6C‐high monocytes and an increased proportion of CD115 + Ly6C‐low monocytes in MD‐1^−/−^ApoE^−/−^ → ApoE^−/−^ chimeric mice compared with control ApoE^−/−^ → ApoE^−/−^ mice, whereas there was no significant difference in the total numbers of monocytes (CD11b + CD115+ subtype cells) (Figure 5A). Compared with ApoE^−/−^ donors, the mRNA expression of pro‐inflammatory markers, including TNF‐α, IL‐6, COX‐2, and iNOs, was decreased in the aorta of MD‐1^−/−^ApoE^−/−^ donors (Figure 5B). By contrast, a robust induction of mRNA related to anti‐inflammatory genes, including PPARγ, Mrc1, IL‐10, and Retnla, was observed in MD‐1^−/−^ApoE^−/−^ → ApoE^−/−^ mice (Figure 5C). Furthermore, the regulatory effects of MD‐1 on the proportion of intraplaque macrophage polarisation tested by double immunofluorescent staining were also elevated. We noticed that iNOs colocalised with CD68 positive cells was decreased in the brachiocephalic arteries or aortic root sections of MD‐1^−/−^ApoE^−/−^ → ApoE^−/−^ mice (Figure 5D,F), whereas a greater abundance of PPARγ expression was found in CD68 positive macrophages in plaques of MD‐1^−/−^ApoE^−/−^ → ApoE^−/−^ mice compared with ApoE^−/−^ mice littermates (Figure 5E,G).

**FIGURE 5:**
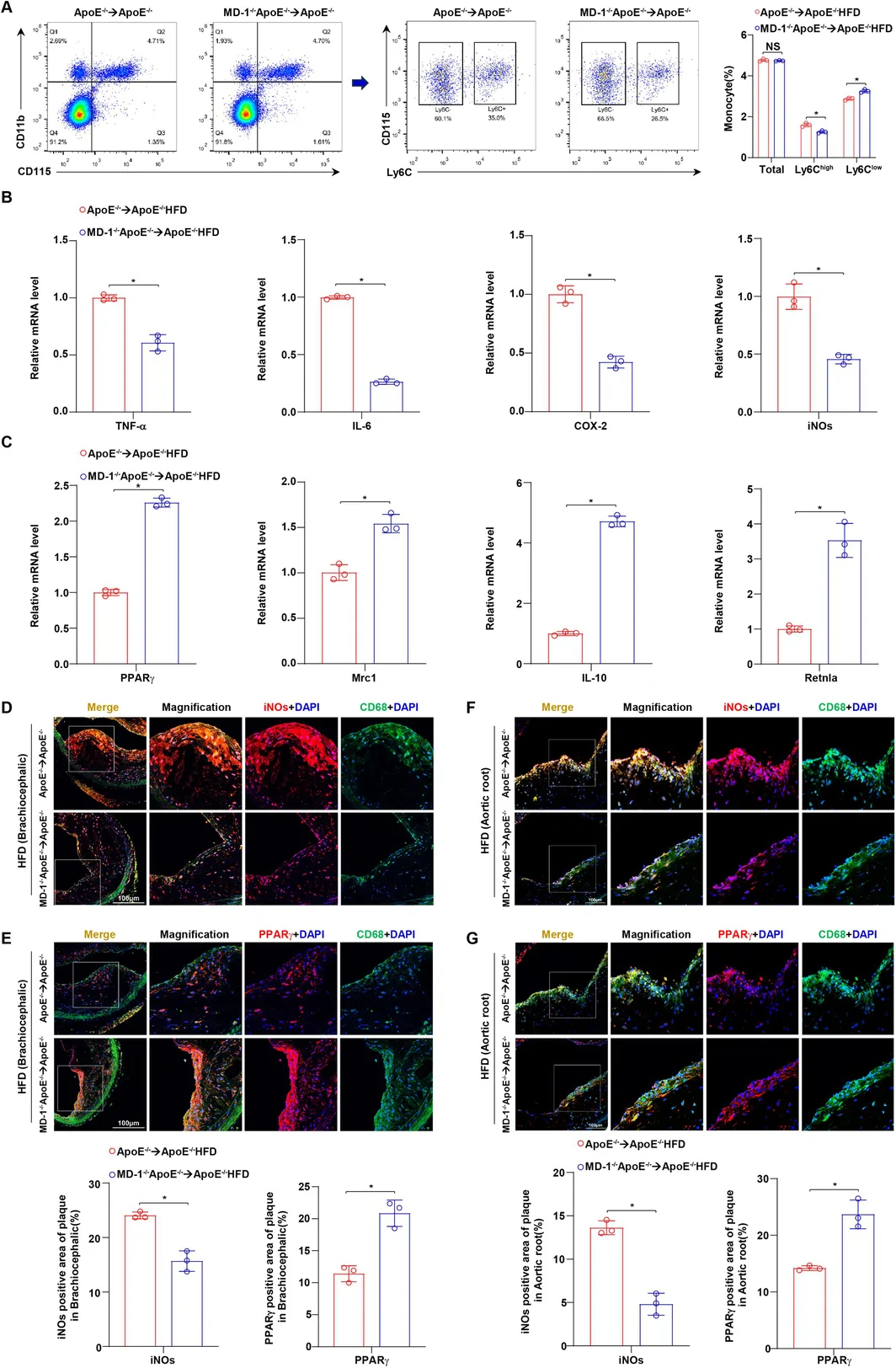
MD‐1 deficiency in macrophage regulates macrophage polarisation in vivo. (A) Total monocytes were gated from live cells as CD11b + CD115+ cells, which were further stratified into Ly6C‐high and Ly6C‐low monocyte subsets based on Ly6C fluorescence intensity. Representative flow plot of mean percentage of CD11b + CD115+, and CD115 + Ly6C‐high or CD115 + Ly6C‐low monocyte in the blood of MD‐1^−/−^ApoE^−/−^ → ApoE^−/−^ mice and ApoE^−/−^ → ApoE^−/−^ mice. *n* = 3. (B, C) RT‐PCR analysis of mRNA expression of TNF‐α, IL‐6, COX‐2 and iNOs for pro‐inflammatory macrophage; PPARγ, Mrc1, IL‐10 and Retnla for anti‐inflammatory macrophage in the aortas of the ApoE^−/−^ mice transplanted with MD‐1^−/−^ApoE^−/−^ or ApoE^−/−^ bone marrow. *n* = 3. (D, E) Immunofluorescence staining for iNOs (red) or PPARγ (red) and CD68 (green) in the brachiocephalic artery sections of the ApoE^−/−^ mice transplanted with ApoE^−/−^ bone marrow or MD‐1^−/−^ApoE^−/−^ bone marrow. *n* = 3. Scale bar: 100 μm. (F, G) Immunofluorescence staining for iNOs (red) or PPARγ (red) and CD68 (green) in the aortic root sections of the ApoE^−/−^ mice transplanted with ApoE^−/−^ bone marrow or MD‐1^−/−^ApoE^−/−^ bone marrow. *n* = 3. Scale bar: 100 μm. *p < 0.05 versus ApoE^−/−^ → ApoE^−/−^ mice treated with HFD group.

### 3.6 MD‐1 Regulates Macrophage Polarisation and Foam Cell Formation In Vitro

Next, the role of MD‐1 in regulating macrophage behaviour was investigated in vitro through MD‐1 loss‐of‐function experiments in bone marrow‐derived macrophages (BMDMs) stimulated with OX‐LDL. Analysis of mRNA expression for macrophage polarisation markers revealed that MD‐1 deletion impaired OX‐LDL‐triggered pro‐inflammatory activation while improving anti‐inflammatory responses. This was characterised by reduced expression of pro‐inflammatory macrophage markers (TNF‐α, IL‐6, COX‐2, and iNOs; Figure 6A) but elevated expression of anti‐inflammatory macrophage markers (PPARγ, Mrc1, IL‐10, and Retnla; Figure 6B) in BMDMs. Furthermore, these polarisation effects resulting from MD‐1 deficiency were confirmed at the protein level by immunoblot analysis (Figure 6C). Notably, reduced lipid accumulation was observed in MD‐1‐deficient BMDMs following OX‐LDL treatment compared to controls (Figure 6D,E), consistent with downregulated expression of the cholesterol uptake gene CD36 and upregulated expression of efflux transporters ABCA1 and ABCG1 [20]. Furthermore, MD‐1 deficiency significantly impaired macrophage migration and proliferation, as evaluated by transwell and BrdU incorporation assays, respectively, and concomitantly reduced the mRNA expression of MMP‐9 and PCNA (Figure 6F–H).

**FIGURE 6:**
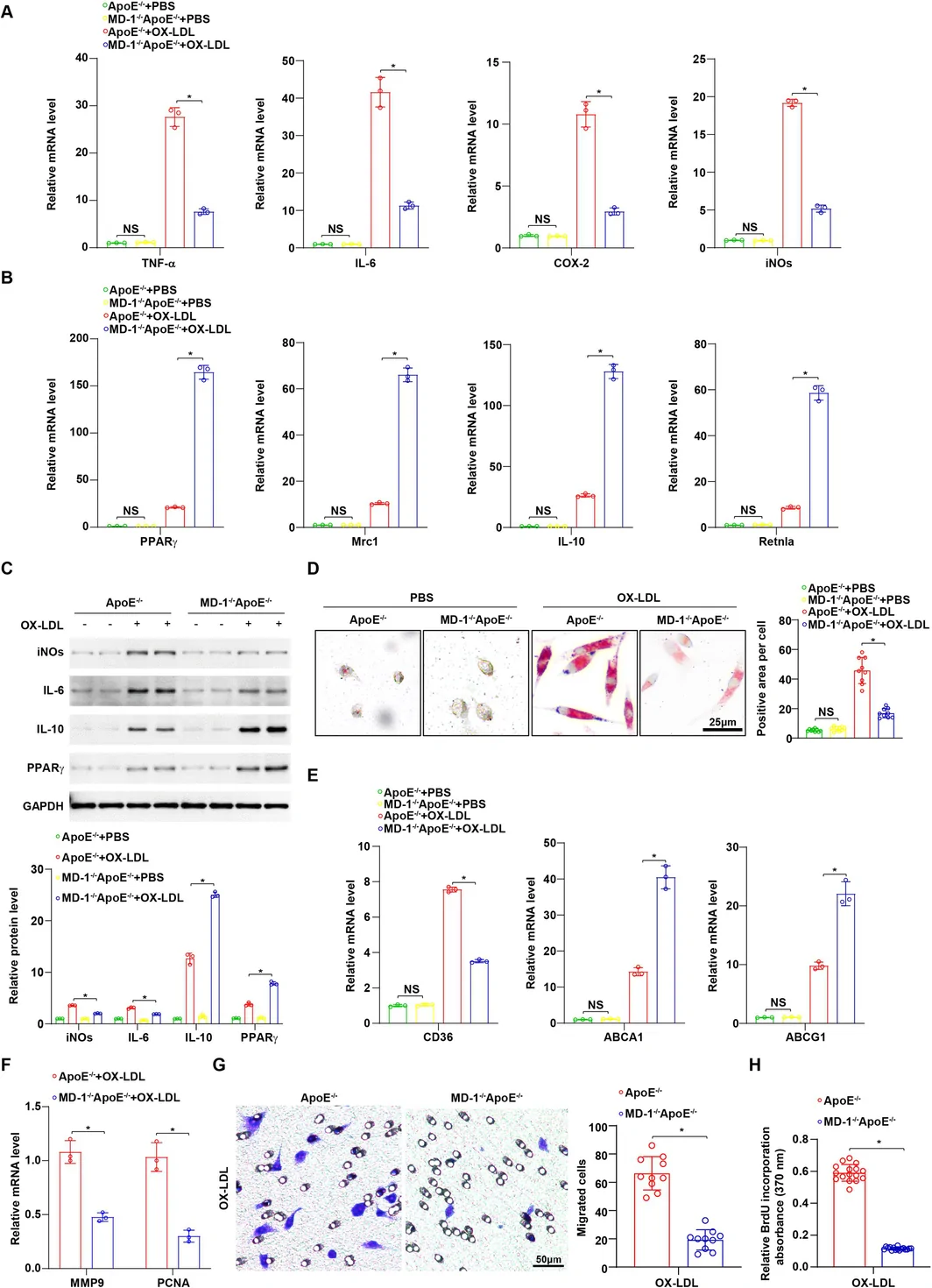
Effects of MD‐1 deficiency on macrophage polarisation and inflammation in vitro. (A, B) RT‐PCR analysis of markers with pro‐inflammatory macrophage (TNF‐α, IL‐6, COX‐2 and iNOs), markers with anti‐inflammatory macrophage (PPARγ, Mrc1, IL‐10 and Retnla) in BMDMs isolated from ApoE^−/−^ or MD‐1^−/−^ApoE^−/−^ mice were incubated with or without OX‐LDL treatment. *n* = 3. (C) Western blot analysis of iNOs and IL‐6, IL‐10 and PPARγ in BMDMs isolated from ApoE^−/−^ or MD‐1^−/−^ApoE^−/−^ mice were incubated with or without OX‐LDL treatment. *n* = 3. (D) The foam cell formation in BMDMs isolated from ApoE^−/−^ or MD‐1^−/−^ApoE^−/−^ mice were incubated with or without OX‐LDL for 24 h followed by oil red O staining. *n* = 10–15. Scale bar: 25 μm. (E) RT‐PCR analysis of CD36, ABCA1 and ABCG1 in BMDMs isolated from ApoE^−/−^ or MD‐1^−/−^ApoE^−/−^ mice were incubated with or without OX‐LDL treatment. *n* = 3. (F) RT‐PCR analysis of migration (MMP9) and proliferation (PCNA) markers of BMDMs isolated from ApoE^−/−^ or MD‐1^−/−^ApoE^−/−^ mice with OX‐LDL administration. *n* = 3. (G) A modified Boyden chamber was performed to analysis the ability of migration of BMDMs isolated from ApoE^−/−^ or MD‐1^−/−^ApoE^−/−^ mice with OX‐LDL administration. *n* = 10. (H) BrdU incorporation was performed to the ability of proliferation of BMDMs isolated from ApoE^−/−^ or MD‐1^−/−^ApoE^−/−^ mice with OX‐LDL administration. *n* = 16. *p < 0.05 versus ApoE^−/−^ → ApoE^−/−^ mice upon OX‐LDL stimulation group.

### 3.7 MD‐1 Deletion Inhibits cGAS‐STING Pathway Regulated NLRP3 Inflammasome Activation

To elucidate the molecular mechanisms underlying MD‐1 deficiency‐mediated macrophage polarisation and atherogenesis, transcriptomic sequencing was performed using BMDMs isolated from MD‐1^−/−^ApoE^−/−^ and ApoE^−/−^ mice following OX‐LDL stimulation (Figure 7A). Gene Ontology (GO) enrichment analysis revealed that differentially expressed genes (DEGs) were significantly associated with inflammatory responses, cytokine activity, oxidative stress responses, lipid metabolic processes, apoptotic regulation, proteolysis, MAP kinase phosphatase activity, and immune responses. Gene heatmaps and gene set enrichment analysis (GSEA) of atherosclerosis‐associated DEGs are presented in Figure 7B–D. Kyoto Encyclopedia of Genes and Genomes (KEGG) pathway analysis indicated significant enrichment of the NOD‐like receptor and NF‐κB signalling pathways (Figure 7E). Notably, differential gene expression analysis demonstrated that NLRP3—a key component of the NOD‐like receptor pathway—was significantly downregulated in the context of MD‐1 deficiency. Given the established role of the NLRP3 inflammasome in inflammatory diseases, including atherosclerosis, protein expression of NLRP3 inflammasome components was examined. Markedly reduced levels of NLRP3‐associated proteins (NLRP3, pro‐IL‐1β, ASC, IL‐1β, and caspase‐1 p10) were observed in OX‐LDL‐treated MD‐1^−/−^ApoE^−/−^ BMDMs (Figure 7F), with a concurrent reduction in NLRP3 colocalisation within macrophages of aortic roots from MD‐1^−/−^ApoE^−/−^ → ApoE^−/−^ chimeric mice (Figure 7G). Because cGAS‐STING activation promotes TBK1‐dependent phosphorylation of IRF3 and NF‐κB, transcription factors that drive pro‐inflammatory cytokine production, we assessed NF‐κB activity. RNA‐seq data confirmed a decrease in NF‐κB transcriptional activity and IRF3 levels in MD‐1‐deficient BMDMs. Collectively, these findings suggest that MD‐1 deficiency attenuates NLRP3 inflammasome activation in atherosclerosis by suppressing the cGAS‐STING‐IRF3/NF‐κB axis. Consistent with the well‐documented pro‐atherosclerotic role of cGAS‐STING signalling, we verified the molecular interaction between MD‐1 and cGAS through multiple validations. We observed the direct in situ interaction between endogenous MD‐1 and cGAS in macrophages tested by Co‐IP and GST pull‐down (Figure 7H,I). High‐resolution confocal immunofluorescence staining in primary BMDMs confirmed prominent intracellular co‐localisation of MD‐1 and cGAS (Figure 7J). Moreover, truncation mutation combined with co‐immunoprecipitation (co‐IP) assays successfully mapped the key functional domains responsible for MD‐1‐cGAS binding (Figure 7K). Furthermore, impaired activation of the cGAS‐STING‐TBK1‐IRF3/NF‐κB pathway was detected in OX‐LDL‐stimulated MD‐1^−/−^ApoE^−/−^ BMDMs (Figure 7L), and MD‐1 binding enhances the enzymatic activity of cGAS to produce cGAMP (Figure 7M), along with reduced STING expression in plaque macrophages of chimeric mice (Figure 7N). To further validate the involvement of cGAS‐STING in MD‐1‐mediated polarisation, BMDMs were treated with the STING agonist DMXAA for 24 h. The decreased expression of TBK1‐dependent phosphorylation of IRF3 and NLRP3‐associated proteins (IL‐1β and caspase‐1 p10) mediated by MD‐1 were reversed by DMXAA (Figure 7O). Functionally, this intervention reversed the MD‐1 ablation phenotype, counteracting both the downregulation of pro‐inflammatory macrophage markers and the upregulation of anti‐inflammatory markers (Figure 7P), as well as foam cell formation and macrophage migration (Figure 7Q,R). Collectively, these results indicate that the athero‐protective effects of MD‐1 knockout on macrophage polarisation are significantly mediated through the suppression of NLRP3 inflammasome activation via the cGAS‐STING pathway.

**FIGURE 7:**
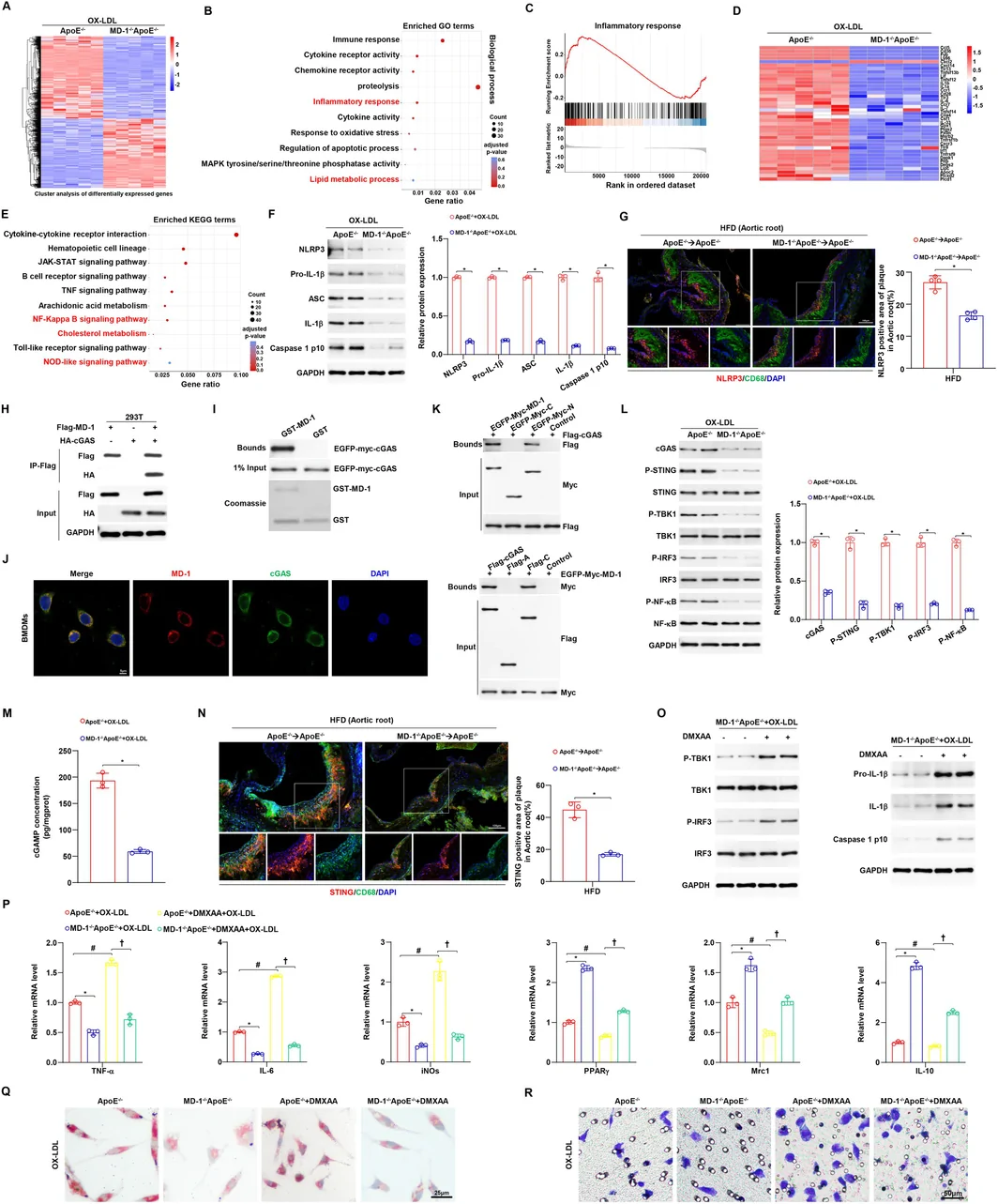
Decreased NLRP3 inflammasome activation mediated by MD‐1 deletion reduced cGAS‐STING pathway. (A) Heatmaps showing that differential gene expression in BMDMs isolated from male ApoE^−/−^ or MD‐1^−/−^ApoE^−/−^ mice upon OX‐LDL administration. (B–E). Gene ontology biological process (GO) enrichment, gene set enrichment (GSE) and KEGG pathway enrichment analysis of the identified DEGs based on RNA‐seq data set from OX‐LDL treated BMDMs isolated from male ApoE^−/−^ or MD‐1^−/−^ApoE^−/−^ mice. The key statistical parameters of GSEA analysis: Normalised enrichment score (NES = 1.50), nominal *p* value = 0.02, FDR *q*‐value = 0.017. (F) Representative western blot images and quantitative analysis of the expression of NLRP3 inflammasome‐associated proteins in BMDMs isolated from ApoE^−/−^ or MD‐1^−/−^ApoE^−/−^ mice with OX‐LDL stimulation. *n* = 3. *p < 0.05 versus ApoE^−/−^ mice upon OX‐LDL stimulation group. (G) Representative immunofluorescence images of NLRP3 (red) and CD68 (green) in the aortic root sections of the ApoE^−/−^ mice transplanted with ApoE^−/−^ or MD‐1^−/−^ApoE^−/−^ bone marrow. *n* = 4. Scale bar: 100 μm. (H) Results from co‐IP assays in 293 T cells transfected with Flag‐tagged MD‐1 and HA‐tagged cGAS. Anti‐Flag and anti‐HA antibodies were used as western blot probes. (I) GST pull‐down assays of enhanced GFP (EGFP)‐Myc‐tagged cGAS with GST or GST‐MD‐1 are indicated. (J) Representative images of MD‐1 (red) colocalised with cGAS (green) in BMDMs. (K) Upper: Mapping the MD‐1‐binding region of cGAS. Cell lysates from HEK293T cells transfected with FLAG‐tagged cGAS and EGFP‐Myc‐tagged MD‐1 deletion mutants were IPed with anti‐FLAG, followed by immunoblotting with anti‐FLAG and anti‐Myc. Below: Mapping the cGAS‐binding region of MD‐1. HEK293T cells were transfected with EGFP‐Myc‐tagged MD‐1 and FLAG‐tagged cGAS deletion mutants. (MD‐1‐FL:1‐162aa, Myc‐C:1–19 aa, Myc‐N:19–162 aa; cGAS‐FL:1‐522 aa, Flag‐A:1‐145 aa, Flag‐C:145‐522aa). (L) Representative western blot images and quantitative analysis of the expression of cGAS, p‐STING/STING, P‐TBK1/TBK1, P‐IRF3/IRF3 and P‐NF‐κB/NF‐κB in BMDMs isolated from ApoE^−/−^ or MD‐1^−/−^ApoE^−/−^ mice after stimulation with OX‐LDL. *n* = 3. *p < 0.05 versus ApoE^−/−^ mice upon OX‐LDL stimulation group. (M) The concentration of cGAMP in BMDMs from ApoE^−/−^ or MD‐1^−/−^ApoE^−/−^ mice treated with OX‐LDL. (N) Representative immunofluorescence images of STING (red) and CD68 (green) in the aortic root sections of the ApoE^−/−^ mice transplanted with ApoE^−/−^ or MD‐1^−/−^ApoE^−/−^ bone marrow. *n* = 3. Scale bar: 100 μm. (O) Representative western blot images of the expression of P‐TBK1/TBK1, P‐IRF3/IRF3, the expression of NLRP3 inflammasome‐associated proteins in BMDMs isolated from MD‐1^−/−^ApoE^−/−^ mice stimulated with DMXAA were subjected to with OX‐LDL. *n* = 3. P‐R. RT‐PCR analysis of TNF‐α, IL‐6, and iNOs for pro‐inflammatory macrophage, PPARγ, Mrc1 and IL‐10 for anti‐inflammatory macrophage; the foam cell formation and migration of BMDMs isolated from ApoE^−/−^ or MD‐1^−/−^ApoE^−/−^ mice stimulated with or without DMXAA, and were subjected to OX‐LDL administration. *n* = 3. Scale bar: 25 μm (Q). Scale bar: 50 μm (R). *p < 0.05 versus ApoE^−/−^ mice upon OX‐LDL stimulation group. ^#^ *p* < 0.05 ApoE^−/−^ mice stimulated with DMXAA versus ApoE^−/−^ mice stimulated without DMXAA upon OX‐LDL stimulation group. ^†^ *p* < 0.05 versus ApoE^−/−^ mice stimulated with DMXAA and upon OX‐LDL stimulation group.

### 3.8 Enhanced MD‐1 and STING/NLRP3 Expression in Human Atheromous Plaque

To further explore whether the expression changes of MD‐1 observed in animal tissues could be recapitulated in human coronary artery plaques, double immunofluorescence assays were performed. Given the limited sample size, the present human tissue analyses were exploratory observational results. Immunofluorescence staining showed that MD‐1 expression was observed to be higher in macrophages of severe human coronary artery plaques (characterised by prominent necrotic cores and thin fibrous caps) than in moderate plaques (featured by intimal hyperplasia with small necrotic cores) (Figure 8A). Similarly, STING and NLRP3 expression was also observed to be higher in the macrophages of severe plaques than in moderate plaques, as detected by double‐immunofluorescence staining (Figure 8B,C). Collectively, these exploratory findings suggest a potential correlation between MD‐1 expression and plaque severity, highlighting the need for further investigation in larger patient cohorts to evaluate its possible role as a biomarker.

**FIGURE 8:**
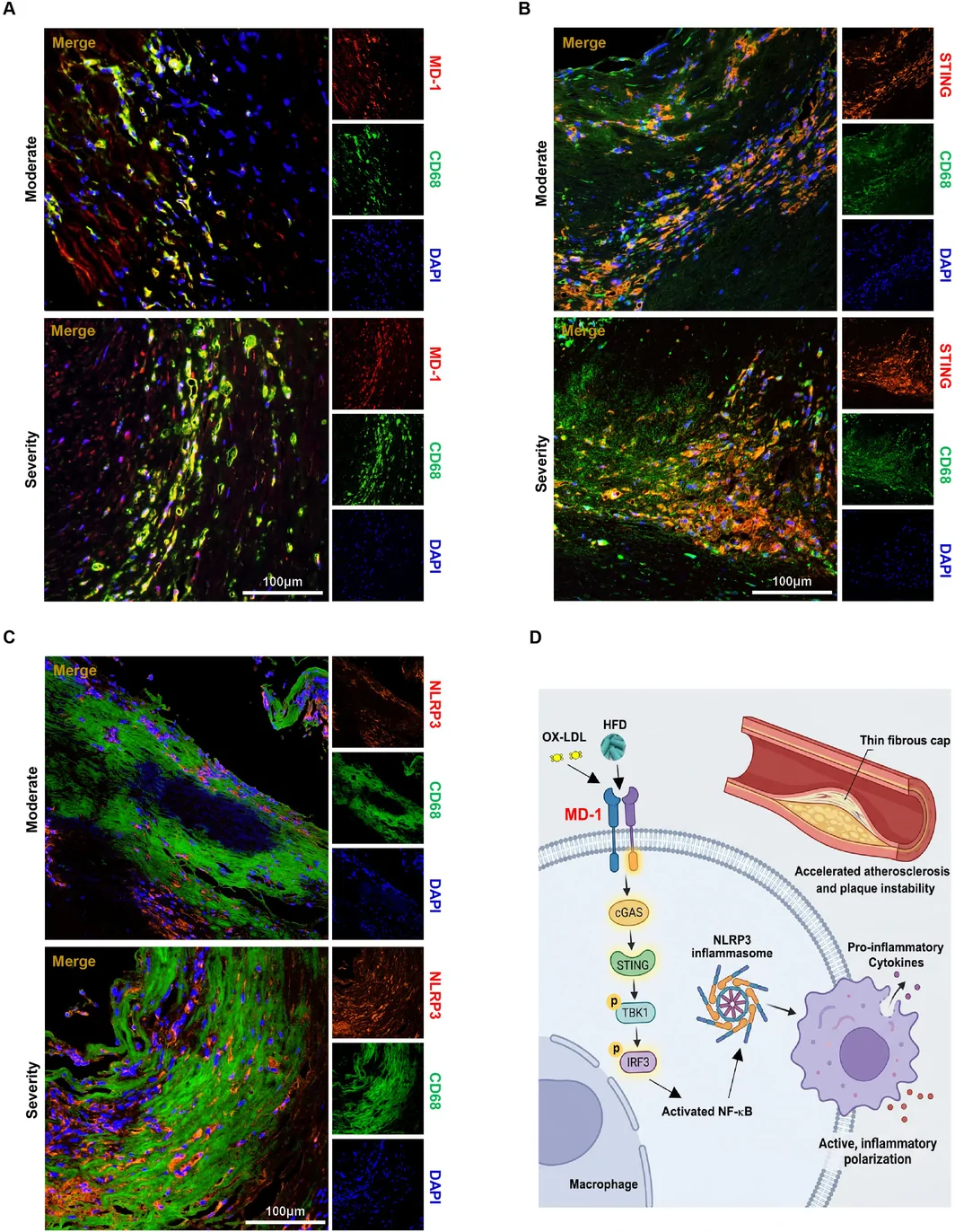
MD‐1 and STING‐NLRP3 expression are upregulated in human atherosclerotic plaque. (A) Immunofluorescence staining for MD‐1 (red) colocalised with CD68 (green) in human tissues with severity or moderate plaque. (B) Immunofluorescence staining for STING (red) colocalised with CD68 (green) in human tissues with severity or moderate plaque. (C) Immunofluorescence staining for NLRP3 (red) colocalised with CD68 (green) in human tissues with severity or moderate plaque. (D) Schematic summary: MD‐1 is induced and activated in macrophages. The activated MD‐1 subsequently promotes cGAS‐STING‐TBK1‐IRF3 signalling pathway to activate NLRP3 inflammasome, and subsequently increase pro‐inflammatory macrophage polarisation and decreased plaque stability to accelerate the development of atherosclerosis.

## 4 Discussion

The crucial cellular effects and molecular mechanisms of MD‐1 on atherogenesis, particularly regarding the regulation of macrophage polarisation, remain poorly investigated. This study highlights the important role of macrophage‐derived MD‐1 in the progression of atherosclerosis, demonstrating its significantly increased expression in atherosclerotic plaques and the loss‐of‐function of MD‐1 with global gene knockout, ablation of MD‐1 in haematopoietic cells with bone marrow transplantation, attenuated pro‐inflammatory macrophage polarisation, and increased plaque stability to protect against the development of atherosclerosis. Mechanistically, MD‐1 deficiency inhibits macrophage polarisation by suppressing the cGAS‐STING‐TBK1‐IRF3 signalling pathway. Collectively, our findings reveal the mechanistic role of macrophage MD‐1 in atherosclerosis and identify novel therapeutic targets (Figure 8D).

Given its ubiquitous expression in metabolic disease, cardiac and vascular tissues [15, 16, 17, 18] and accumulating clinical and preclinical evidence linking MD‐1 expression to carotid atherosclerosis and plaque stability, MD‐1 has recently emerged as a critical regulator of cardiovascular and metabolic diseases [21, 22]. Combining protein–protein interaction network analysis, RT‐PCR, immunohistochemistry, and Western blotting, previous studies have confirmed upregulated MD‐1 expression in ligated mouse carotid arteries [13], while differential gene expression analyses of human carotid atherosclerotic specimens have verified elevated MD‐1 levels in atherosclerotic lesions correlating with inflammatory injury, tissue necrosis, adverse clinical outcomes, and poorer prognosis associated with higher MD‐1 expression [14]. Mechanistically, neutrophil extracellular traps (NETs) amplify local vascular inflammation to drive carotid plaque instability. A documented pathogenic feedback loop couples MD‐1, NET formation, and plaque destabilisation: increased MD‐1 in patients with unstable carotid plaques facilitates NET production via TLR signalling, while excess NETs reciprocally upregulate MD‐1 in macrophages to worsen plaque inflammatory injury [23]. In line with these clinical and pathological observations, our GEO‐database analysis and in vivo experiments further validate the pro‐atherosclerotic role of MD‐1. MD‐1 was significantly upregulated in human atheroma and advanced atherosclerotic plaques; likewise, hyperlipidemic ApoE deficient mice showed markedly elevated aortic MD‐1, predominantly localised to plaque macrophages. Functional assays revealed that MD‐1 exacerbated vascular inflammation and accelerated carotid plaque progression, consistent with our observation that genetic MD‐1 ablation in ApoE^−/−^ mice reversed vulnerable‐plaque features, limited lesion formation, and enhanced plaque stability. Subsequent bone‐marrow transplantation experiments further confirmed that macrophage‐derived MD‐1 was a key atherosclerosis regulator: macrophage‐specific MD‐1 deficiency suppressed pro‐inflammatory macrophage polarisation, promoted anti‐inflammatory phenotypic switching, and mitigated atherosclerotic progression. These findings support the central contribution of macrophage polarisation to atherogenesis [8, 24] and identify MD‐1 as a critical modulator of macrophage functional reprogramming in atherosclerosis.

Elevated MD‐1 expression has been reported in multiple vascular pathological injuries [13, 14], including carotid atherosclerosis, consistent with our findings in aortic plaques. Despite anatomical, hemodynamic, and lesion‐progression differences between the aorta and carotid arteries, MD‐1 confers a conserved pro‐atherosclerotic effect in both vascular territories via regulating innate immune signalling and macrophage activation, indicating that MD‐1‐driven pro‐inflammatory signalling constitutes a common pathogenic mechanism across different arterial segments in atherosclerosis. Collectively, our data, together with previous evidence, firmly establish MD‐1 as a key pro‐atherogenic factor in both aortic and carotid plaques. Further investigations focusing on carotid lesions are required to clarify potential tissue‐specific regulatory features of MD‐1. The present study adopted multiple experimental models, including global MD‐1‐knockout mice, bone‐marrow transplantation, and in vitro primary BMDM cultures, to dissect MD‐1 function in atherosclerosis. One limitation of our bone‐marrow transplantation model is that MD‐1 is deleted in all haematopoietic lineages instead of being macrophage‐specific. Given that MD‐1 has been reported to modulate neutrophil NET formation and neutrophil‐mediated vascular inflammation [23], the atheroprotective phenotypes observed in chimeric mice may result from the combined effects of multiple haematopoietic cell types, rather than macrophage‐specific MD‐1 deletion alone. Future studies utilising LysM‐Cre‐mediated macrophage‐specific conditional MD‐1 knockout mice will be essential to precisely define the cell‐autonomous function of macrophage MD‐1 in atherogenesis.

To uncover the downstream molecular mechanism by which macrophage‐derived MD‐1 modulates macrophage polarisation and atherosclerotic progression, we performed RNA sequencing on OX‐LDL‐stimulated BMDMs isolated from MD‐1^−/−^ApoE^−/−^ and control ApoE^−/−^ mice. KEGG pathway enrichment analysis identified NOD‐like receptor and NF‐κB signalling pathways as the core signalling cascades modulated by MD‐1. Further differential expression analysis confirmed that MD‐1 deficiency significantly suppressed the expression of NLRP3, the central component of the NLRP3 inflammasome. The NLRP3 inflammasome is a cytosolic multi‐protein complex composed of NLRP3, ASC, and caspase‐1, which drives atherosclerotic inflammation by promoting caspase‐1 activation, pro‐IL‐1β and pro‐IL‐18 cleavage, and the maturation and secretion of pro‐inflammatory IL‐1β and IL‐18. Sustained NLRP3 inflammasome activation is widely recognised as a pivotal driver of atherosclerotic lesion progression and plaque destabilisation [25, 26, 27]. Consistent with these transcriptomic results, MD‐1‐deficient BMDMs displayed pronounced downregulation of NLRP3 inflammasome‐associated proteins, and reduced NLRP3 expression was also validated in aortic root macrophages of MD‐1^−/−^ApoE^−/−^ chimeric mice. As a canonical upstream priming signal for NLRP3 inflammasome activation, NF‐κB signalling triggers the transcription of NLRP3, IL‐1β, and IL‐18 following nuclear translocation. Subsequent stimulation by ATP, pore‐forming toxins, and reactive oxygen species further induces NLRP3 inflammasome assembly and full activation, thereby facilitating the maturation and release of IL‐1β and IL‐18 and exacerbating vascular inflammatory injury [28, 29].

Beyond the canonical NF‐κB‐NLRP3 axis, our study identifies the cGAS‐STING pathway as a key upstream mediator of MD‐1‐driven atherogenesis. The cGAS‐STING cascade is an evolutionarily conserved cytosolic DNA‐sensing system governing innate immunity and inflammation [30, 31, 32]. Aberrant cGAS‐STING activation in atherosclerotic macrophages aggravates foam‐cell generation, inflammatory infiltration and plaque instability by driving M1‐proinflammatory polarisation and suppressing M2‐anti‐inflammatory polarisation via STAT6 signalling and downstream inflammatory mediators [33, 34, 35, 36, 37, 38]. Consistent with its established pro‐atherosclerotic function, we validated molecular interaction between MD‐1 and cGAS using multiple assays. High‐resolution confocal immunofluorescence revealed robust intracellular co‐localisation of MD‐1 and cGAS in primary BMDMs. GST pull‐down further confirmed direct endogenous MD‐1‐cGAS binding in macrophages, and truncation‐mutant co‐immunoprecipitation (co‐IP) mapped their critical binding domains. Notably, MD‐1 is conventionally regarded as a secreted/membrane bound glycoprotein, whereas cGAS is cytosolic, the potential mechanistic explanations for this phenomenon including: First, a fraction of newly synthesised MD‐1 may undergo intracellular retention in macrophages due to saturation of the secretory pathway or alternative splicing variants lacking a signal peptide, thereby enabling its transient physical contact with cytosolic cGAS before complete secretion or membrane trafficking. Second, membrane‐anchored MD‐1 could be internalised via endocytic pathways under certain conditions. Upon cellular stress or endosomal maturation, these vesicles might fuse with lysosomes or undergo membrane rupture, allowing MD‐1 to gain access to the cytosol or endomembrane compartments where cGAS resides. Third, within the pathological microenvironment of atherosclerotic plaques, macrophages are frequently exposed to mechanical strain, oxidative stress, and inflammatory mediators, which may lead to transient plasma membrane permeabilisation or micro‐injury. Under such circumstances, extracellular MD‐1 could translocate across the damaged membrane and interact with cytosolic cGAS. Further investigations are warranted to dissect the precise subcellular topology and trafficking dynamics governing the MD‐1–cGAS association in distinct cellular states. Functional experiments showed that MD‐1 deficiency strongly repressed cGAS‐STING‐TBK1‐IRF3‐NF‐κB signalling in aortic macrophages, accompanied by reduced STING abundance and diminished NF‐κB transcriptional activity. Importantly, STING agonist DMXAA largely reversed the M2 biased anti‐inflammatory phenotype triggered by MD‐1 knockout, verifying that MD‐1 regulates macrophage polarisation and atherosclerotic progression in a STING dependent manner.

Nevertheless, the precise molecular mechanisms by which MD‐1 regulates STING signalling, including potential transcriptional regulation, post‐transcriptional modification, and remodelling of STING‐associated protein complexes, remain to be systematically elucidated. The TLR4/NF‐κB pathway represents another classic innate inflammatory cascade driving atherosclerotic inflammation, and extensive crosstalk between TLR4 and cGAS‐STING signalling has been well documented in innate immune cells. Although we cannot fully exclude potential crosstalk with the TLR4/NF‐κB pathway, our findings support that MD‐1‐mediated regulation of macrophage‐dependent atherogenesis relies primarily on the cGAS‐STING cascade. Further investigations are required to dissect the reciprocal regulatory relationship between MD‐1 and TLR4 signalling. In addition, while DMXAA‐based pharmacological rescue partially reversed the inflammatory and phenotypic abnormalities of MD‐1‐deficient macrophages, the current study lacks specific genetic or pharmacological rescue experiments targeting TBK1, IRF3, NF‐κB, and NLRP3. This limitation precludes a definitive conclusion regarding the indispensable roles of these downstream mediators in MD‐1‐dependent regulatory effects. Notably, comparable serum lipid levels were observed between MD‐1 knockout and control mice, indicating that MD‐1 promotes atherosclerosis predominantly by inducing local vascular inflammation rather than modulating systemic lipid metabolism. Although previous studies have linked STING and NLRP3 inflammasome activity to circulating lipid regulation, our data reveal a tissue‐specific pro‐inflammatory role of MD‐1 in vascular lesions. Future investigations focusing on lipoprotein oxidation and lipid clearance will help fully clarify the crosstalk among MD‐1, inflammatory responses, and lipid metabolism during atherogenesis.

Clinical sample analysis demonstrated that the expression levels of MD‐1, STING, and NLRP3 in plaque macrophages were positively correlated with the severity of human atherosclerotic lesions, reinforcing the clinical translational significance of our mechanistic findings. Collectively, our results define a novel pathogenic cascade in atherosclerosis: macrophage‐derived MD‐1 amplifies the cGAS‐STING‐NF‐κB signalling axis, thereby potentiating NLRP3 inflammasome activation and subsequent vascular inflammatory responses. Nevertheless, we acknowledge a key limitation of the present study: the observed clinical correlations cannot establish causal relationships. It remains unclear whether elevated MD‐1 expression actively drives atherosclerotic progression or merely serves as a secondary inflammatory biomarker of lesion inflammation. Large‐scale prospective clinical cohort studies and Mendelian randomisation analyses are therefore warranted to distinguish the causal pathogenic role of MD‐1 from its passive biomarker function in human atherosclerosis.

In summary, our findings reveal that MD‐1 ablation exerts a previously unrecognised athero‐protective effect by attenuating the development of atherosclerosis through the regulation of macrophage polarisation. We further elucidate a novel molecular mechanism: MD‐1 activates the cGAS‐STING signalling pathway, promoting the activation of the NLRP3 inflammasome and subsequent secretion of pro‐inflammatory cytokines. Our findings highlight the critical function of macrophage MD‐1 in atherogenesis and provide promising novel therapeutic targets for atherosclerotic cardiovascular diseases.

## Author Contributions

W.‐L.C., X.H. and Z.L. conceived and designed the experiments. F.Z., Z.J. and Z.C. performed the experiments. Q.Z., T.H. and X.K. analysed and interpreted the data. M.C. and X.H. contributed reagents, materials, analysis tools or data. F.Z. did the analysis. W.‐L.C. wrote the paper. All authors reviewed the manuscript.

## Funding

This work was supported by grants from the Yellow Crane of Excellence Program (HHYC2019003), Zhongnan Hospital of Wuhan University Science, Technology and Innovation Seed Fund (CXPY2023034) and Translational Medicine and interdisciplinary Research Joint Fund of Zhongnan Hospital of Wuhan University (ZNJC202405).

## Conflicts of Interest

The authors declare no conflicts of interest.

## Data Availability

The data that support the findings of this study are available from the corresponding author upon reasonable request.
